# Knowledge vs. Action: Discrepancies in University Students' Knowledge about and Self-Reported Use of Self-Regulated Learning Strategies

**DOI:** 10.3389/fpsyg.2017.01288

**Published:** 2017-07-27

**Authors:** Nora M. Foerst, Julia Klug, Gregor Jöstl, Christiane Spiel, Barbara Schober

**Affiliations:** Educational Psychology and Evaluation, Department of Applied Psychology: Work, Education, Economy, University of Vienna Vienna, Austria

**Keywords:** self-regulated learning, higher education, knowledge, action, transfer, competences, university, production deficiency

## Abstract

University students are supposed to be autonomous learners, able to adapt to an educational environment significantly less guided than school. Entering higher education poses a challenge of self-regulation, in which beginning students are often not prepared with self-regulation strategies needed. Since there are many studies assessing self-regulated learning (SRL) via classical self-reports, we know a lot about how students generally self-assess their SRL strategies. However, SRL and performance do not always correlate highly in these studies. The aim of the present study is to determine whether there are discrepancies between students' knowledge about SRL and their action in applying adequate SRL strategies in relevant learning situations. We also want to know whether such discrepancies generalize across domains and what the reasons for discrepancies are. The situation-specific Self-Regulated Learning Questionnaire for Action and Knowledge (SRL-QuAK) was used in a sample of 408 psychology and economic sciences students. Descriptive data analysis was conducted to determine potential discrepancies between SRL knowledge and action and differences between the study domains in an explorative way. The reasons for not using SRL-strategies were derived via qualitative content analysis. The results showed that although students had quite advanced knowledge of SRL strategies, they did not put this knowledge into action. This dissonance between SRL knowledge and action was found in both domains. In terms of reasons, students stated that they (a) lacked the time to use SRL strategies, (b) would not benefit from SRL strategies in the given situation, (c) would not be able to put the strategies to use effectively or (d) found it too arduous to use SRL strategies. The implications of these results will be discussed, e.g., the consequences for measures to overcome students' dissonance between knowledge and action and therefore to promote academic performance and well-being.

## Introduction

University students are typically assumed to be autonomous learners able to adapt to a rather unguided educational environment. University students have to deal with diverse challenges (Wild, 2000; Streblow and Schiefele, 2006), including, for example, planning and organizing their own learning, self-motivation for learning and dealing with emotions. As Dresel et al. (2015) point out, higher education is characterized by complex achievement tasks and great autonomy with respect to learning organization, materials, collaboration, learning goals, content and procedures, as well as relatively few opportunities to receive external feedback. Hence, a very important skill for students to develop in order to be successful in higher education is self-regulated learning (SRL). SRL refers to “self-generated thoughts, feelings, and actions that are planned and cyclically adapted to the attainment of personal goals” (Zimmerman, 2000, p. 14). Self-regulated students are able to adapt their cognition, behavior, motivation and emotion to the goals they set for themselves. SRL is not only a goal of curricular frameworks (European Commission, 2008), but also a relevant topic in educational research (e.g., Zimmerman and Schunk, 2011). It is considered a prerequisite for academic success (e.g., Boekaerts, 1997; Wirth and Leutner, 2008), and meta-analyses of SRL interventions have indicated substantial effects on students' achievement (e.g., Hattie et al., 1996; Dignath et al., 2008). There is a large body of literature on SRL (for overviews, see Boekaerts et al., 2000; Zimmerman and Schunk, 2011) as well as different approaches to measuring SRL (for overviews see Boekaerts and Corno, 2005; Wirth and Leutner, 2008; Zimmerman, 2008). Although there have been several waves of SRL measurement in which various measures were developed and used (Panadero et al., 2016), a large share of studies still rely on classical self-report questionnaires (Roth et al., 2016). Typical biases like socially desirable answers or self-serving biases need to be taken into account in these classical self-assessment studies. Furthermore, as most studies still rely on classical self-report questionnaires, we know little about students' knowledge regarding beneficial and adverse strategies in relevant learning situations in higher education as well as students' actual usage of SRL strategies in these situations, since classical questionnaires do not ask about these topics. In line with research on production deficits (Hasselhorn and Gold, 2006) and difficulties transferring learned content into practice (e.g., Haskell, 2001; Day and Goldstone, 2012), one could assume that even if students know a lot about SRL strategies, there could still be interferences when it comes to applying these strategies in real-life higher education situations. Thus, the aim of the present study is to obtain some initial insights into students' actual use of beneficial and adverse SRL strategies in relevant higher education situations and how it relates to their knowledge about which strategies are beneficial or adverse. Specifically, we want to know if there are discrepancies between students' knowledge and action with regard to SRL strategies in higher education. Since SRL strategies are not domain-specific (Dörrenbächer and Perels, 2016) and can be applied to a wide range of domains (Núñez et al., 2011), we also want to know if possible discrepancies generalize across different domains. Most importantly, we want to know what reasons students give for the discrepancy between knowledge and action in order to improve students' use of SRL strategies by targeting the reasons students report for not applying beneficial strategies.

### Self-regulated learning as a competence

Although SRL has been extensively researched, a number of definitions and theoretical models coexist (e.g., Pintrich and Garcia, 1994; Boekaerts, 1999; Zimmerman, 2002; Schmitz and Wiese, 2006). If we want to get insight into students' actual use of beneficial and adverse SRL strategies in relevant higher education situations and how it relates to their knowledge about which strategies are beneficial or adverse, we have to rely on the components as well as the SRL process summarized in the prominent models of self-regulated learning for designing new measures with which we can answer our questions. However, we have to address these strategies in a more comprehensive way, addressing more recent conceptualizations of SRL as a competence and overcoming some disadvantages of commonly used instruments. Zimmerman (2002) already stated that SRL is not a mental ability or an academic performance skill. It is also not a trait or personal characteristic. If it would be, it would be difficult to change or to train, and it probably would be stable across situations and domains. In the course of research innovations in competence modeling and measuring, Wirth and Leutner (2008) conceptualized and defined SRL as a competence. Competences in general have been described as “context-specific cognitive dispositions that are acquired and needed to successfully cope with certain situations or tasks in specific domains” (Koeppen et al., 2008, p. 62). Recently, competences have been conceptualized on a continuum between knowledge and performance, with performance as the manifestation of competence (compare Blömeke et al., 2015). Consequently, competencies in SRL go beyond strategy knowledge. They also embody the application of such strategies in a way that is fruitful for performance and fits the challenging situations that are relevant in a specific domain. The conceptualization of SRL as a competence underlines the importance of learning more about students' knowledge as well as action with regard to SRL strategies. Furthermore, domain, context and situation specificity should be addressed when measuring SRL knowledge and action in an innovative way.

### Measurement of self-regulated learning

Analogous to the large number of SRL conceptualizations, there is also a large number of measurement approaches to SRL (Boekaerts and Corno, 2005). Wirth and Leutner (2008) categorize them into offline and online standards and quantitative and qualitative standards. Offline standards aim to assess components of SRL, whereas online standards aim to monitor processes. Online standards change depending on the conditions and context of the learning situation (see Wirth and Leutner, 2008), which is in line with the conceptualization of SRL as a context- and situation-specific competence. According to Wirth and Leutner (2008), quantitative standards assume that applying more SRL strategies comprises an improvement in SRL, whereas qualitative standards assume that the fit between the strategies applied and the specific learning situation and task is crucial for being a good self-regulated learner. Thus, qualitative standards are again more in line with the conceptualization of SRL as a competence. Dresel et al. (2015) also point out the relevance of focusing on the fit between the specific self-regulatory demands of a learning situation and the applied strategies. However, in a recent review of SRL measures, Roth et al. (2016) point out that the most commonly used measures are still standard self-report questionnaires that fit the category of quantitative offline standards. These measures are mainly used for economical reasons because their administration is easy for both participants and researchers, even in large-scale assessments (Cromley and Azevedo, 2011). Nonetheless, although the significance of SRL as a competence has become quite well-known, there are still evident deficits in its assessment. Classical self-report questionnaires serve us well for the mentioned economical reasons if we want to go for large-scale assessments, but they have up to now applied quantitative offline standards. There are still no questionnaires that are easy to administer to large samples but at the same time take situation and context specificity into account. Furthermore, there are no questionnaires as of yet that ask about students' actions in relation to knowledge, and finally, students' reasons for not applying beneficial SRL strategies against their better judgment have not been assessed empirically as of yet. Getting this kind of information, even from a questionnaire, could help us improve study programs and promote the actual application of SRL strategies.

### The present empirical investigation

In the present empirical investigation, we aim to obtain deeper insight into university students' knowledge and action concerning SRL strategies in an explorative way. To do so, we use an innovative measurement approach that follows rather qualitative online standards than quantitative offline standards by taking conditions and context of learning situations as well as the fit between strategies applied and the specific learning situation into account. However, it still is in the form of a questionnaire that is easy to administer, analyze and interpret. By using this innovative measure, we get different information than we get by using common questionnaires, since it differentiates between knowledge and action. It addresses the challenges of measuring SRL economically but still in a situation- and context-specific way. Moreover, we get information about reasons for possible discrepancies between knowledge and action against one's better judgment, which, in turn, help us in designing more effective interventions. The study was part of a broader multi-method-multi-informant joint research project addressing the explicit need to triangulate measurements for approaching SRL as a competence (as suggested by Boekaerts and Corno, 2005; Panadero et al., 2016). The project involved four universities that investigated SRL in four different fields of study in order to explore the application of SRL in different domains (mathematics, psychology, engineering and economic sciences). In order to take into account the complexity of SRL as a competence that cannot be captured by a single measurement approach, four different instruments (situation-specific questionnaire, situational judgment test, learning diary, and e-portfolio) representing either process- or product-oriented measures were developed. For this study, the situation-specific questionnaire (Self-Regulated Learning Questionnaire for Action and Knowledge, SRL-QuAK) was used to gain insight into knowledge and action regarding beneficial and adverse strategies as well as reasons for discrepancies between knowledge and action across different domains, psychology and economic sciences in this case.

To summarize, the desiderata, which mainly stem from the drawbacks of the classical measurement of SRL, are the following: There is little knowledge so far on how much students know about beneficial and adverse SRL strategies in relevant higher education situations and whether they actually put these strategies into practice. In line with that, we do not know as of yet whether there are discrepancies between students' knowledge and action when it comes to SRL strategies. Additionally, since SRL strategies are not domain-specific, but can be applied across a wide range of domains, it is also important to know whether the patterns between knowledge and action are similar in different domains in order to take adequate measures. Lastly and most importantly, also with regard to taking adequate measures, we do not know as of yet what reasons keep students from using beneficial SRL strategies against their better judgment. Consequently, we address the following research questions in this paper:

1. Are there discrepancies between students' knowledge about beneficial and adverse SRL strategies and their actual usage of beneficial and adverse SRL strategies?
2. If discrepancies between knowledge and action are found, are they generalizable across different domains or are they domain-specific?
3. If there are discrepancies, which reasons do students report for not applying beneficial strategies against their better judgment?

Answering these questions can help us promote students' SRL competences in an advanced and more specific way by addressing reasons for possible discrepancies between knowledge and action.

## Methods

### Sampling and procedure

The sample of the present study was comprised of *N* = 408 students at the University of Vienna (224 female, 93 male, 91 not stated; age: *M* = 25.36 years, *SD* = 4.88). To achieve comparability between domains as well as in study progress (Bachelor's vs. Master's/Diploma programs), the sub-samples consisted of 175 (43%) students of psychology and 233 (57%) students of economic sciences. 226 (55%) were enrolled in a Bachelor's program, 89 (22%) in a Master's program, and 93 (23%) in a diploma program in one of the two fields of study. The students were recruited via formal email requests to participate in the study in the winter semester of 2015. The e-mail contained a link to an online questionnaire created in unipark (www.unipark.de). Attention was drawn to the study invitation to participate in courses and lectures. To minimize the effort for participants, the questionnaire was split into four sub-questionnaires related to four different situations with corresponding relevant SRL strategies that had previously been found to be important in higher education (Dresel et al., 2015). To maintain comparability between sub-questionnaires, the students were randomly assigned to one of the four sub-questionnaires via unipark. Each sub-questionnaire took about 25 min to finish.

### Measure

As the initial point for developing the SRL-QuAK qualitative expert interviews and quantitative expert ratings were conducted and (a) typical situations demanding SRL in university studies, (b) relevant SRL strategies for these situations during university studies, and (c) the fit between SRL situations and corresponding SRL strategies were identified (see Dresel et al., 2015). Experts were asked to assess the benefit of each SRL strategy class for different learning situations. The results indicated metacognitive strategies to be highly relevant when writing a minor academic paper, cognitive strategies when preparing for an exam, strategies of boredom regulation when attending a lecture and strategies of frustration regulation when writing a major scientific thesis. The typical situations served as vignettes for the questionnaire introducing the given situation (e.g., writing a minor academic paper; see Appendix A). A short description of the formal challenges contained in the situation (e.g., researching literature, writing, and submitting an academic text by a given deadline) was presented and the participants were asked to put themselves in the situation described.

Since we aimed to analyze domain-generalizability, the four vignettes are not domain-specific, but applicable to all study programs. The SRL-QuAK comprises four sub-questionnaires, each introduced via one of the vignettes describing a relevant learning situation identified by experts (compare Dresel et al., 2015) as requiring (1) metacognitive, (2) cognitive, (3) frustration or (4) boredom regulation strategies.

The vignettes were followed by SRL strategies that were either beneficial or adverse (distractors) in the given situation. Participants had to consider whether each strategy would be beneficial or adverse in the situation described in the vignette, drawing on their general declarative knowledge about the benefit of a specific strategy in a given situation (e.g., setting goals when writing a major scientific thesis). By contrast, the procedural level related to knowledge about what sub-strategies are contained within a strategy (know-how; e.g., including breaks and buffer time into a schedule, cf. Paris et al., 1983). Again, students had to decide whether a sub-strategy related to how to implement a given strategy more concretely would be beneficial or adverse in the situation described in the vignette. Thus, each procedural knowledge scale (assessing one relevant sub-strategy) corresponds to a single item in the declarative knowledge scale. The procedural level measure consisted of items derived and adapted from renowned self-report questionnaire scales such as the LIST (Wild and Schiefele, 1994) and SVF-KJ (Coping questionnaire for children and adolescents; Hampel et al., 2001), as well as theory-based (e.g., Pekrun, 2006; Streblow and Schiefele, 2006) self-constructed items. The situation-specific strategies were validated via quantitative expert ratings (*N* = 11) by renowned German-speaking scholars in the field of SRL research. The experts were presented with the vignettes and asked to state for each item (on declarative and procedural level) whether they deem it beneficial or adverse in the given situation of the vignette. To determine the reliability of agreement between the experts on the binary scale ratings, κ/*SE*_(κ)_ (Fleiss, 1971) was calculated and tested against a minimum acceptable level of agreement, by calculating a confidence interval (CI). The analysis of the inter-rater agreement from the expert rating resulted in κ = 0.73 and κ/*SE*_(κ)_ = 23.10 (α = 0.05). The two-sided 95% CI_(κ)_ = [0.69–0.78] indicates a substantial agreement (Landis and Koch, 1977).

**Figure 1:**
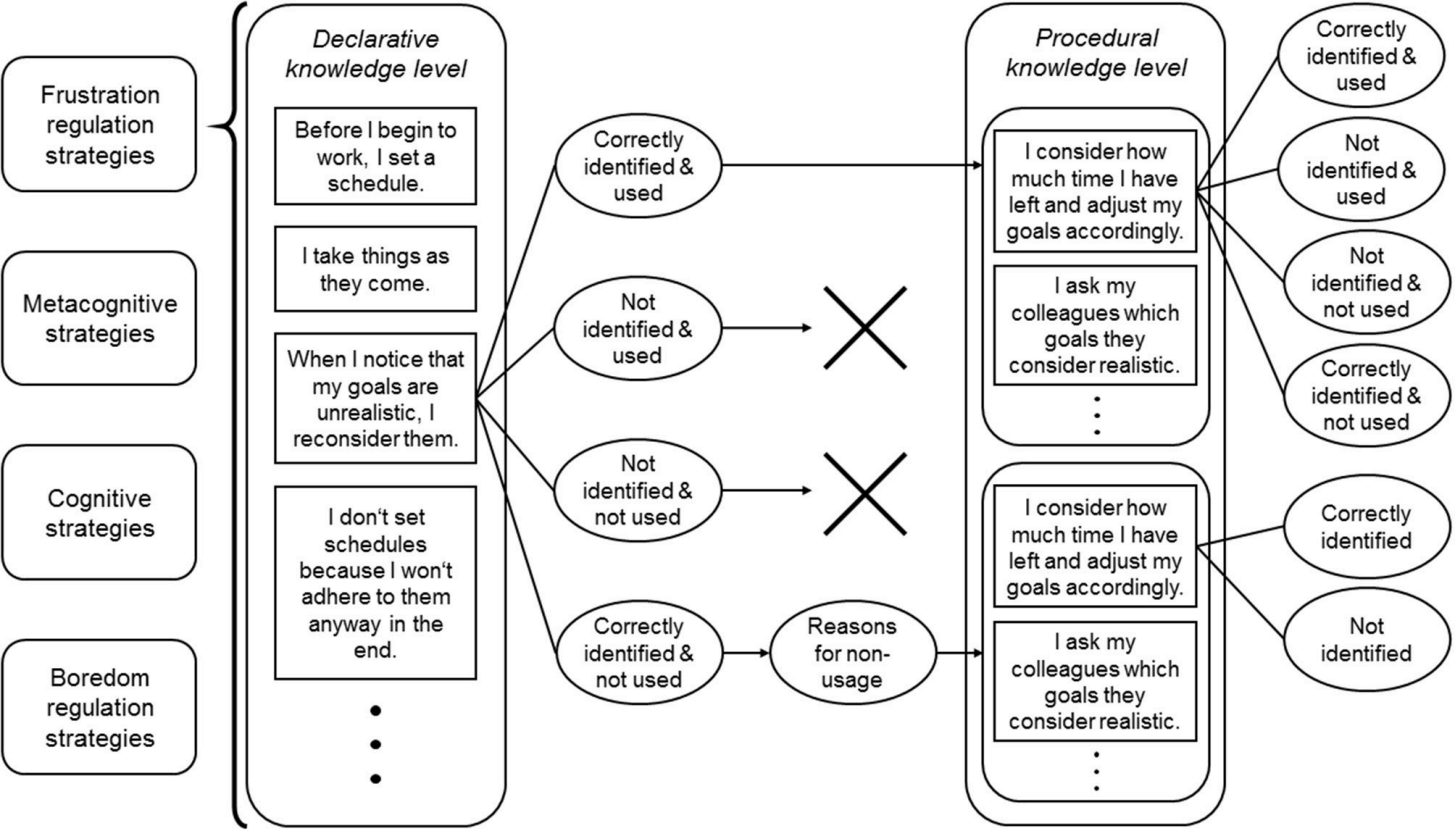
The adaptive structure of the questionnaire using frustration regulation strategies as an example. If the strategy on the declarative knowledge level is adverse, then one proceeds to the procedural knowledge level, when the strategy is correctly identified as adverse and not used or when it is correctly identified and used.

Each item on the declarative and procedural level consisted of a statement that required an answer in a dichotomous response format (“yes” or “no”) to the questions “Is this beneficial?” and “Do you use this strategy?,” with the latter representing the action component of SRL-QuAK. The questionnaire followed an adaptive structure in which procedural knowledge and action were addressed only if students identified the corresponding beneficial SRL-strategies on the declarative level (see Figure 1). Furthermore, if students assessed a certain SRL strategy as beneficial, but reported not putting it into practice, they were asked about the reason for this discrepancy between knowledge and action via an open answer format, to gain insight into the mechanisms that prevent students from using a specific strategy.

### Analyses

To get an explorative insight into whether there are discrepancies between students' knowledge about beneficial and adverse SRL strategies and their actual usage of beneficial and adverse SRL strategies, descriptive statistical analyses were conducted using SPSS Version 22. Descriptive statistics for all items were calculated for the knowledge and the usage dimension separately, for both beneficial and adverse strategies. The descriptive statistics were calculated for the overall sample as well as the sub-samples of psychology and economic sciences students, which can be compared descriptively in order to check exploratory for generalizability across different domains.

Moreover, we checked inference statistically for the discrepancies between knowledge and action in both domains as well as for the generalizability of the knowledge and action pattern across domains. To do so, we differentiated between beneficial and adverse strategies and coded if students identified them correctly as beneficial or adverse. We aggregated over all beneficial and similarly over all adverse items for knowledge and action in each of the four strategy dimensions (metacognitive, cognitive, frustration and boredom regulation strategies), which we tested separately. In order to check for discrepancies, we calculated two indices for each strategy dimension: a discrepancy index for beneficial and a discrepancy index for adverse strategies. The discrepancy index for beneficial strategies was calculated by subtracting the relative ratio of applied beneficial strategies from the relative ratio of correctly identified beneficial strategies. The discrepancy index for adverse strategies was calculated by subtracting the relative ratio of not applied adverse strategies from the relative ratio of correctly identified adverse strategies. Positive values in the discrepancy indices show us that knowledge is higher than action. In order to test if the discrepancies differ statistically significant from zero, we calculated one sample *t*-tests with the discrepancy indices for beneficial and adverse strategies for each strategy dimension. Statistically significant *t*-values show us that the discrepancies differ statistically significant from zero, thus indicating discrepancies between knowledge and action. In order to test for the generalizability of knowledge and action patterns between the two tested domains, we calculated two-sample *t*-tests for group differences between economic science and psychology students. If there was a group difference in a discrepancy index between economic science and psychology students in a strategy dimension, the one-sample *t*-tests were calculated separately for each domain. Homoscedasticity was given for all analyses.

For research question 3, which focuses on the open answers about reasons for discrepancies between knowledge and action, we used a qualitative content analytic approach that combined inductive and deductive steps, using MAXQDA 12. First, via inductive reasoning, categories of reasons were formed on the basis of students' aggregated open answers. Second, answers were deductively assigned to the categories and the most frequently reported categories of reasons were identified. Moreover, two independent raters rated a random sample of 20% (232) of the open answers to the four most common categories in order to check for inter-rater reliabilities using Cohen's Kappa (see Section Results).

## Results

### University students' knowledge and self-reported use of SRL strategies

**Figure 2:**
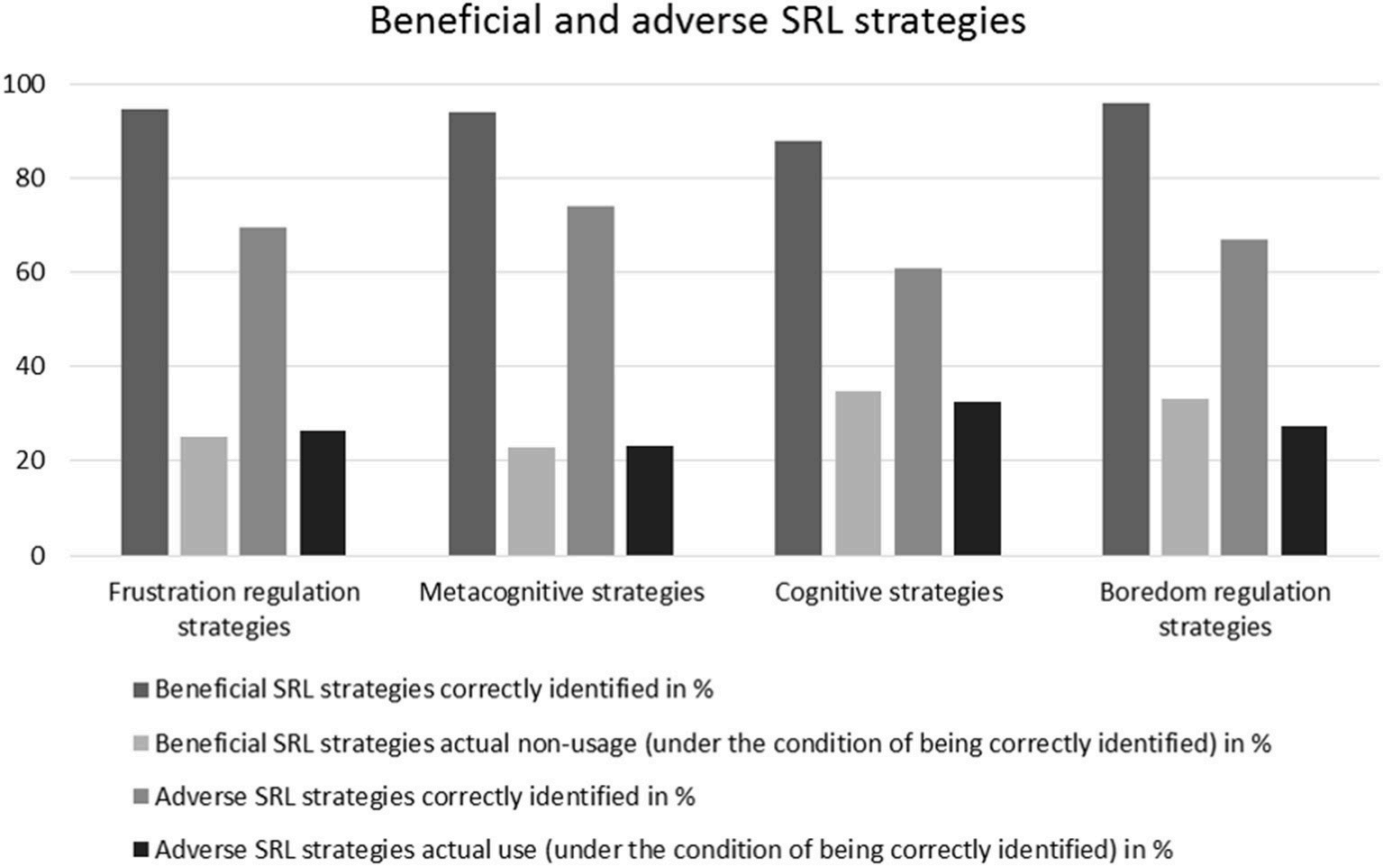
Means of correctly identified beneficial SRL strategies and actual non-usage of beneficial SRL strategies vs. means of correctly identified adverse SRL strategies and actual use of adverse SRL strategies.

In order to answer research question (1), whether a differential analysis of SRL competence—in the sense of looking at both knowledge and usage of SRL strategies—can provide more information than a general competence factor, we compared the rates of correct identification of SRL strategies (beneficial vs. adverse) and the non-usage of beneficial strategies/usage of adverse strategies (see Figure 2).

The analysis of the overall sample showed high rates of correct identification of beneficial SRL strategies, ranging from *M* = 87.7% (*SD* = 10.3) in the sub-questionnaire “Cognitive strategies” to *M* = 95.8 (*SD* = 4.2) in “Boredom regulation strategies.” Still, the actual non-usage of the correctly identified SRL strategies ranges from *M* = 22.8% (*SD* = 12.0) in “Metacognitive strategies” to *M* = 34.8% (*SD* = 18.0) in “Cognitive strategies.” For an overview of university students' knowledge and self-reported use of beneficial SRL strategies, see Table 1. When it comes to adverse SRL strategies, the rates of correct identification range from *M* = 60.9% (*SD* = 15.1) in “Cognitive strategies” to *M* = 73.9% (*SD* = 16.1) in “Metacognitive strategies.” The percentage of actual use of correctly identified adverse strategies ranges from 23.2% (*SD* = 10.2) in “Metacognitive strategies” to 32.4% (*SD* = 13.7) in “Cognitive strategies.” For an overview of university students' knowledge and self-reported use of adverse SRL strategies, see Table 2.

**Table 1:** Overall means and standard deviations of correctly identified beneficial SRL strategies and actual non-usage in the overall sample.

**Beneficial SRL strategies**	**Correctly identified in %**				**Actual non-usage in % (under the condition of being correctly identified)**			
	***M***	***SD***	**Min**	**Max**	***M***	***SD***	**Min**	**Max**
Frustration regulation strategies	94.7	4.8	81.8	100	25.0	12.0	11.3	56.8
Metacognitive strategies	94.0	6.5	68.1	100	22.8	12.0	1.6	53.4
Cognitive strategies	87.7	10.3	57.3	99.2	34.8	18.0	10	79.8
Boredom regulation strategies	95.8	4.2	86.6	100	33.2	23.1	2.8	80.4

*The minima and maxima are on item level. The minimum of actual non-usage in % can be interpreted as the lowest discrepancies between knowledge and action, the maxima as the highest discrepancies*.

**Table 2:** Overall means and standard deviations of correctly identified adverse SRL strategies and actual use in the overall sample.

**Adverse SRL strategies**	**Correctly identified in %**				**Actual use in % (under the condition of being correctly identified)**			
	***M***	***SD***	**Min**	**Max**	***M***	***SD***	**Min**	**Max**
Frustration regulation strategies	69.6	19.6	40.5	92.6	26.4	14.6	10.5	49.3
Metacognitive strategies	73.9	16.1	26.5	94.2	23.2	10.2	5.7	44.1
Cognitive strategies	60.9	15.1	33.9	88.5	32.4	13.7	8.1	50.8
Boredom regulation strategies	66.9	23.8	31.6	94.6	27.3	17.4	6.5	58.1

*The minima and maxima are on item level. The minimum of actual non-usage in % can be interpreted as the highest discrepancies between knowledge and action, the maxima as the lowest discrepancies*.

In order to test inference statistically for the discrepancies between knowledge and action, we calculated one sample *t*-tests for beneficial and adverse strategy discrepancy indices in each of the four strategy dimensions. In each strategy dimension for beneficial as well as for adverse strategies, *t*-values were positive and reached statistical significance [beneficial metacognitive strategies *t*_(146)_ = 17.17, *p* < 0.001; adverse metacognitive strategies *t*_(146)_ = 8.41, *p* < 0.001; beneficial boredom regulation strategies *t*_(93)_ = 13.47, *p* < 0.001; adverse boredom regulation strategies *t*_(93)_ = 8.34, *p* < 0.001; beneficial frustration regulation strategies *t*_(146)_ = 16.61, *p* < 0.001; adverse frustration regulation strategies *t*_(146)_ = 6.21, *p* < 0.001; beneficial cognitive strategies *t*_(122)_ = 17.39, *p* < 0.001; adverse cognitive strategies *t*_(122)_ = 4.17, *p* < 0.001]. The statistically significant positive *t*-values mean that the discrepancy indices all differ statistically significant from zero, indicating higher knowledge than action.

### Generality across domains

**Figure 3:**
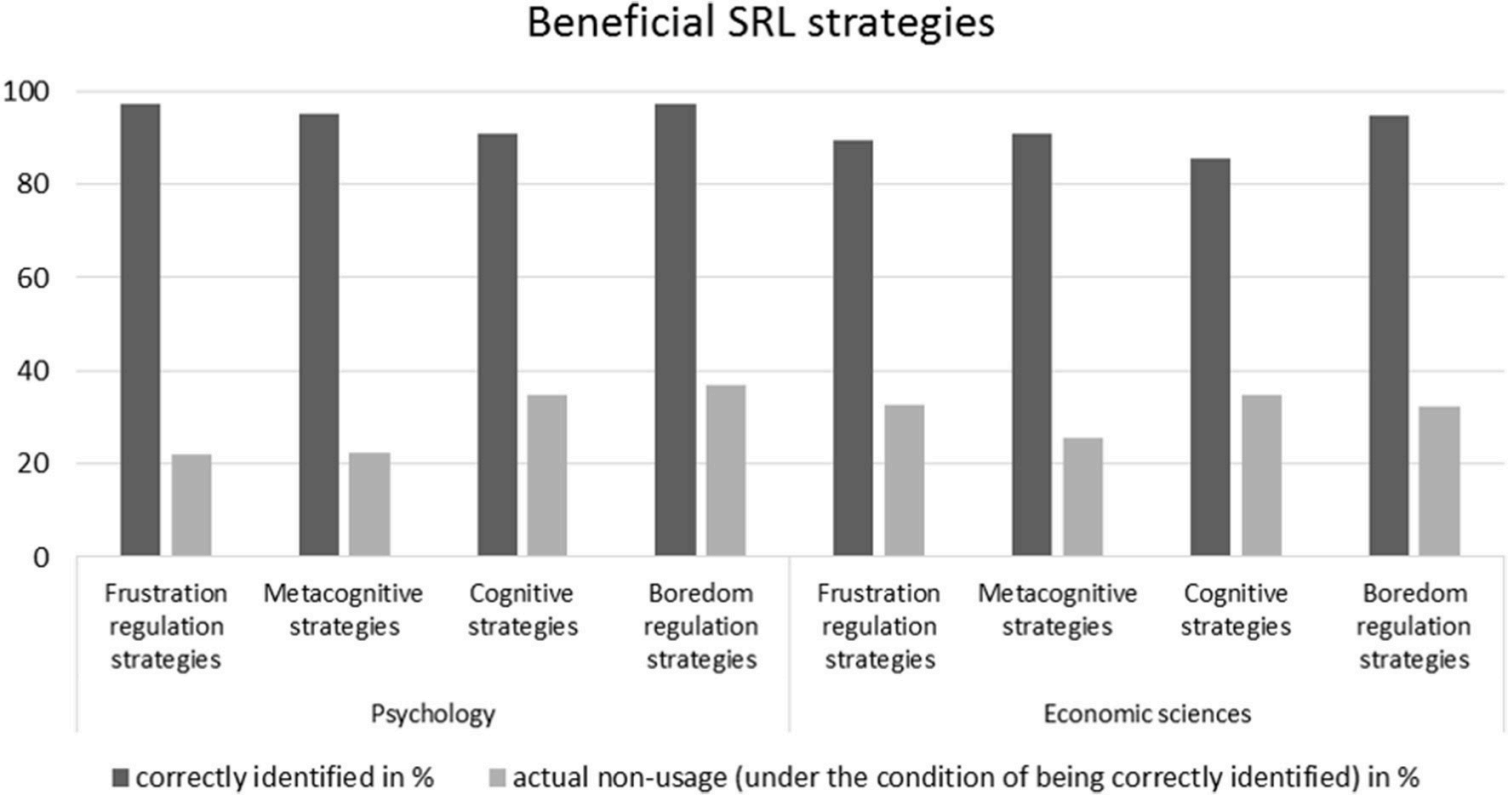
Means of correctly identified beneficial SRL strategies and actual non-usage in the sub-samples of economic sciences and psychology.

**Figure 4:**
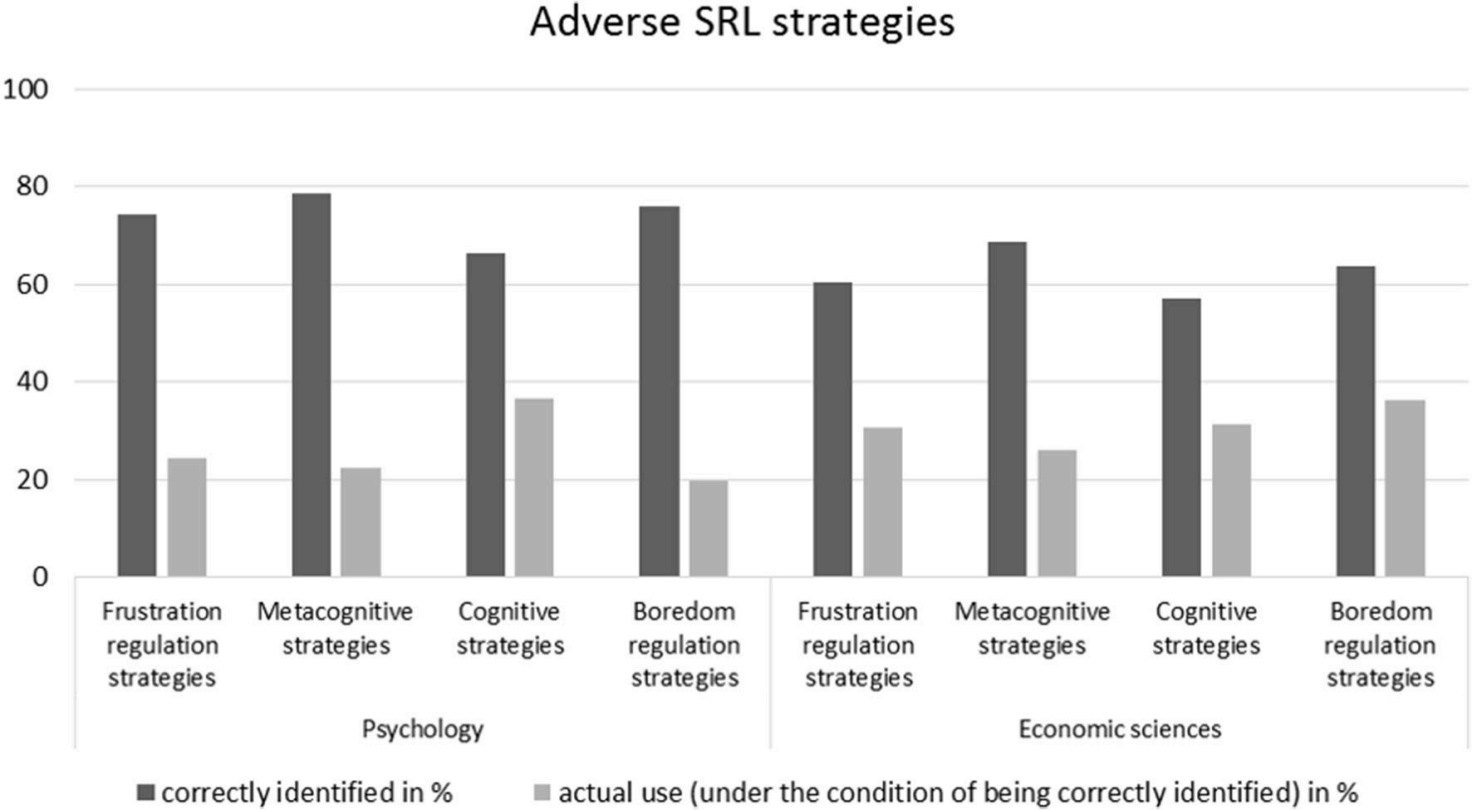
Means of correctly identified adverse SRL strategies and actual use in the sub-samples of economic sciences and psychology.

In order to answer research question (2), whether the discrepancies between knowledge and action described above can be found not only for a specific study program but are generalizable across domains, we analyzed the distributions of knowledge and action concerning beneficial (see Figure 3) and adverse SRL strategies (see Figure 4) separately for students of economic sciences and psychology.

Directly comparing the two domains of psychology and economic sciences descriptively shows that the pattern of discrepancies between knowledge and action concerning SRL strategies is similar across different domains. It is to be noted that psychology students exhibit descriptively higher rates of correct identification of both beneficial and adverse SRL strategies than students of economic sciences. The picture becomes even more differentiated when it comes to the application of beneficial SRL strategies (see Table 3): Whilst students of economic sciences report using slightly more metacognitive (*M* = 25.5%, *SD* = 13.8) and considerably more frustration regulation (*M* = 32.6%, *SD* = 15.5) strategies than psychology students (*M* = 22.4%, *SD* = 12.3 and *M* = 22.1%, *SD* = 11.7), the latter state a slightly higher use of boredom regulation strategies (*M* = 37%, *SD* = 27.6 compared to *M* = 32.3%, *SD* = 23.1). The use of cognitive strategies is quite balanced between the sub-samples (*M* = 34.8%, *SD* = 19.8 and *M* = 34.9%, *SD* = 20.0). When it comes to adverse SRL strategies (see Table 4), it's striking that the samples report quite similar use of adverse strategies and beneficial strategies. Comparing the sub-samples, students of economic sciences report higher uses of adverse SRL strategies, with the exception of adverse cognitive strategies (*M* = 31.3%, *SD* = 15.6), for which psychology students attain a higher overall mean (*M* = 36.3%, *SD* = 19.5).

**Table 3:** Overall means and standard deviations of correctly identified beneficial SRL strategies and actual non-usage in the sub-samples of economic sciences and psychology.

**Adverse SRL strategies**	**Fields of study**			
	**Economic sciences**		**Psychology**	
	**Correctly identified M in % (*SD*)**	**Actual non-usage M in % (*SD*)**	**Correctly identified M in % (*SD*)**	**Actual non-usage M in % (*SD*)**
Metacognitive strategies	90.8 (7.9)	25.5 (13.8)	95.3 (6.6)	22.4 (12.3)
Cognitive strategies	85.4 (9.6)	34.8 (19.8)	91.0 (13.7)	34.9 (20.0)
Boredom regulation strategies	94.8 (5.3)	32.3 (23.1)	97.1 (4.6)	37.0 (27.6)
Frustration regulation strategies	89.5 (8.4)	32.6 (15.5)	97.4 (3.5)	22.1 (11.7)

**Table 4:** Overall means and standard deviations of correctly identified adverse SRL strategies and actual use in the sub-samples of economic sciences and psychology.

**Adverse SRL strategies**	**Fields of study**			
	**Economic sciences**		**Psychology**	
	**Correctly identified M in % (*SD*)**	**Actual use M in % (*SD*)**	**Correctly identified M in % (*SD*)**	**Actual use M in % (*SD*)**
Metacognitive strategies	68.6 (20.9)	26.0 (19.0)	78.7 (14.9)	22.2 (11.0)
Cognitive strategies	57.0 (16.3)	31.3 (15.6)	66.5 (15.9)	36.3 (19.5)
Boredom regulation strategies	63.6 (26.3)	36.1 (21.7)	76.1 (20.6)	19.8 (20.9)
Frustration regulation strategies	60.3 (19.9)	30.5 (15.4)	74.4 (19.4)	24.5 (15.0)

In order to test inference statistically for the generalizability of knowledge and action patterns between the two tested domains, we calculated two-sample *t*-tests for group differences between economic science and psychology students. In most cases, there were no statistically significant group differences in the discrepancy indices [beneficial metacognitive strategies *t*_(145)_ = −0.84, *p* = 0.40; adverse metacognitive strategies *t*_(145)_ = −1.65, *p* = 0.10; beneficial boredom regulation strategies *t*_(92)_ = −0.41, *p* = 0.69; adverse boredom regulation strategies *t*_(92)_ = 0.10, *p* = 0.92; beneficial cognitive strategies *t*_(121)_ = −0.80, *p* = 0.43; adverse frustration regulation strategies *t*_(145)_ = −1.77, *p* = 0.08], indicating that the pattern of discrepancies between knowledge and action is similar for economic science and psychology students. There were statistically group differences between economic science and psychology students in the discrepancy indices in two cases, namely beneficial frustration regulation strategies [*t*_(145)_ = 3.11, *p* < 0.01] and adverse cognitive strategies [*t*_(121)_ = −2.97, *p* < 0.01]. For beneficial frustration regulation strategies, the discrepancy index is higher for economic science students, indicating a bigger discrepancy between knowledge and action in economic science. When we calculated one-sample *t*-tests separately for beneficial frustration regulation strategies in each domain, we still found statistically significant discrepancies for economic science students [*t*_(47)_ = 10.10, *p* < 0.001] as well as for psychology students [*t*_(98)_ = 14.00, *p* < 0.001]. For adverse cognitive strategies, the discrepancy index is higher for psychology students, indicating a bigger discrepancy between knowledge and action in psychology. When we calculated one-sample *t*-tests separately for adverse cognitive strategies in each domain, we still found statistically significant discrepancies for psychology students [*t*_(48)_ = 5.97, *p* < 0.001], but not for economic science students [*t*_(73)_ = 1.34, *p* = 0.18].

### Reported reasons for discrepancies between knowledge and action

The 13 reason categories (see Table 5) were generated based on a mixed deductive and inductive approach. In a first step, one rater identified the main themes of the open answers. These themes were then discussed and adapted by the research team, taking into account the attributional theory of achievement motivation and emotion (Weiner, 1985) as well as Expectancy × Value Theory (Heckhausen, 1977). In a next step, two other raters coded the open answers according to these 13 plus one categories. Overall, 1,159 open answers were classified into 13 reason categories plus one “not codable” category. The four categories most frequently reported were a) I lack the time to use this strategy (*f* = 628); (b) I'm not able to use this strategy effectively (*f* = 239); (c) I don't see the benefit of this strategy, considering the situation/task (*f* = 229); and (d) Using this strategy would be too arduous (*f* = 190). Choosing four categories as a cut-off point resulted from deductive content considerations on the basis of Expectancy × Value Theory (Heckhausen, 1977) and attributional theory (Weiner, 1985). The inter-rater reliability of coding a random sample of 20% (232) of the open answers proved to be sufficient, with Cohen's Kappa being κ = 0.77 for the sub-questionnaire “Frustration regulation strategies,” κ = 0.90 for the sub-questionnaire “Cognitive strategies,” κ = 0.71 for the sub-questionnaire “Boredom regulation strategies” and κ = 0.73 for the sub-questionnaire “Metacognitive strategies.” Looking at the sub-questionnaires separately (see Table 6), the reason code “I'm not able to use this strategy effectively” was the most stated reason in “Frustration regulation strategies” and “Boredom regulation strategies,” whilst “I don't see the benefit of this strategy, considering the situation/task” was the most frequent answer in “Metacognitive strategies” and “I lack the time to use this strategy” in “Cognitive strategies.”

**Table 5:** Coding frequencies of the reason categories and corresponding coding frequency percentage.

**Reason categories**	**Frequency**	**Percentage**
Not codable	668	25.4
I lack the time to use this strategy	628	23.9
I'm not able to use this strategy effectively	239	9.1
I don't see the benefit of this strategy, considering the situation/task	229	8.7
Using this strategy would be too arduous	190	7.2
I'm not interested in the topic	155	5.9
I don't see the benefit of this strategy	124	4.7
The surrounding conditions do not allow this strategy to be used	93	3.5
I can put this strategy into practice, effectively	75	2.9
The study program requires me to fulfill certain formalities	69	2.6
It's not worth it.	56	2.1
I don't dare to	52	2.0
I'd rather invest my time in other things	35	1.3
The study program does not promote the use of this strategy	16	0.6
Total	2,629	100

**Table 6:** Coding frequencies of the four reason categories for two raters of a 20% random sample of open answers.

**Sub-questionnaire**	**Coding frequencies**			
	**Lacking the time to use the SRL strategy**	**Not seeing the benefit of the SRL strategies in the given situation**	**Not being able to put the strategies to use effectively**	**Finding it too arduous to use SRL strategies**
Frustration regulation strategies	17	20	56	20
Metacognitive strategies	65	85	35	37
Cognitive strategies	95	28	14	13
Boredom regulation strategies	35	32	57	41
Total	212	165	162	111

## Discussion

The aim of this study was to obtain a more differentiated view of what students know about SRL vs. what they actually do as well as a first insight into the reasons that prevent students to use SRL strategies despite their better judgment.

Our first point of interest was whether differentiating between knowledge about SRL and usage of SRL could provide additional information on SRL as a competence. We found the same trend of discrepancies between knowledge and action for both beneficial and adverse SRL strategies. However, the adverse strategies had a lower rate of correct identification, which poses the question of why students believe adverse strategies to actually be beneficial more often than vice versa. One reason for this may lie in the structure of study programs: From the beginning of their university studies, students must complete a number of tests and exams, quickly receiving grades that serve as a baseline for them to establish a set of strategies that will allow them to achieve their academic goals whilst maintaining an economical cost-value ratio. For multiple choice tests—which are most frequently used in the early university years at the University of Vienna—this means that quite often students are rewarded for using surface processing learning strategies, since a multiple choice answering format mainly draws on direct recognition. Thus, it's very possible that students learn that strategies considered adverse for any goal beyond mere recognition are actually very economical in terms of their cost-value ratio. However, study program requirements intensify in subsequent semesters, leaving students overwhelmed by challenges that cannot be tackled with the previous set of strategies. This would also tie in with the finding that psychology students—despite showing a tendency for higher rates of correct SRL strategy identification—show higher discrepancies when it comes to adverse cognition strategies, meaning that they put adverse cognitive strategies (mainly surface processing strategies) to use, despite being able to identify them as adverse.

However, it's also plausible that the adverse strategies were harder to recognize, since the challenge during item development was to create items that possessed sufficient item difficulty—in the sense of not making it too easy to differentiate between beneficial and adverse strategies—in order to obtain differential information about students' knowledge of SRL.

Although the rate of correctly identified strategies is very high for both sub-samples, psychology students show higher rates when it comes to beneficial strategies for all sub-questionnaires and noticeably higher identification rates for adverse strategies than economic science students. In an attempt to understand this tendency, we examined the content of both study programs on a curricular level: Beyond the aim of imparting professional expertise, the psychology curriculum states that students should gain a form of meta-learning expertise. The Bachelor program includes a seminar that explicitly seeks to impart competences in time and knowledge management as well as reflective handling of professional expertise and individual competences. In the Master program, self-regulation is an explicit focus in one of the three specializations offered. No equivalent is found in the economic sciences curriculum, which might explain why economic sciences students showed higher discrepancies between knowledge and action in group comparison for beneficial frustration regulation strategies. The integration of SRL into the curriculum could explain the psychology students' greater knowledge in this field, but this fact also highlights the lack of a corresponding effect on the actual use of these strategies. Although the analyses showed slight differentiation in the extent, the same pattern of discrepancies between knowledge and action of SRL strategies are prominent in both sub-samples, indicating a generalizability of SRL, as inherent to the concept of defining SRL as a competence.

In the literature, different categories of deficiencies have been identified as posing possible challenges in the process of implementing SRL strategies (Hasselhorn and Gold, 2006; Spörer and Brunstein, 2006; Nückles and Wittwer, 2014). The “mediation deficiency” describes a state where learners do not yet possess the cognitive preconditions to effectively put a SRL strategy into practice. In concordance to our questionnaire design, Spörer and Brunstein (2006) characterize this level as not being able to identify a SRL strategy. Interestingly, this ties in with one of the most often reported reasons for non-usage of SRL strategies in our study, “I'm not able to use this strategy effectively.”

Despite our expectation that students had already learned about SRL strategies, the students often stated that the presented learning situation vignettes (e.g., writing a scientific thesis) were new to them and they did not know how to approach this challenge yet. Looking at the stated reasons in dependence of the sub-questionnaire, we find that this mediation deficiency seems to appear mostly in resource-oriented strategy classes, namely boredom and frustration regulation. This could be attributed to the lack of strategy impartation that focuses on affective regulation in students. Whilst cognitive and meta-cognitive strategies have a more self-evident connection to student's learning outcomes, resource-oriented strategies might easily be overlooked as powerful mediators.

On the other hand, students with “production deficiencies” possess the cognitive prerequisites for using a relevant SRL strategy, but have not yet incorporated it into their common behavior repertoire and hence do not spontaneously put it to use effectively. In a questionnaire format, this would be operationalized by learners being able to identify a strategy correctly but not putting it into practice autonomously (see Spörer and Brunstein, 2006). This deficiency might be most crucial for understanding the discrepancy between SRL knowledge and action. The underlying reasons for this deficiency have already been addressed theoretically, as it is presumed that the spontaneous usage of SRL strategies requires a readiness to do so, which can be confounded by a lack of willingness to learn or lack of meta-strategic knowledge (Nückles and Wittwer, 2014). In accordance with these proposed reasons, we found that many students stated that “using [an SRL] strategy would be too arduous” (indicating deficient willingness) and “[I] don't see the benefit of this strategy, considering the situation/task” (indicating a lack of meta-strategic knowledge about which strategy can be useful in what situation).

Whilst attributed to the same deficiency category, these two reasons imply entirely different challenges: Insufficient willingness is predicated on motivational variables and an unpropitious cost-value ratio. A lack of meta-strategic knowledge, on the other hand, can be addressed by imparting more than declarative knowledge about SRL strategies. Supporting this theory even more so, and “[I] don't see the benefit of this strategy, considering the situation/task” was the most often stated reason in the sub-questionnaire “Metacognitive strategies,” whilst “I lack the time to use this strategy” was the most frequently stated reason for “Cognitive strategies.” As mentioned above, we must consider the structure and demands of university curricula. If multiple choice tests are the exam mode of choice, the costs of using beneficial cognitive strategies that result in a deeper understanding stand in no relation to the demands of recognition that this mode requires.

Overall, the results clearly indicate that we must shift our focus from the mere transfer of declarative knowledge to the promotion of procedural knowledge and most of all, practice of SRL strategies.

### Limitations and implications for research and practice

Although SRL is deemed to be essential for academic success, studies have not been able to attribute a substantial proportion of the variance in academic performance to SRL (see Schiefele et al., 2003). The reason for this may be rooted in conceptual or methodological issues. This study represents a first step toward a new, differentiated measurement approach. Even if the SRL-QuAK still depends to a certain part on self-report, it includes some features that are different to usual self-report questionnaires. For example, it is situation-specific and asks for beneficial and adverse strategies in specific situations. Thus, it rather relies on qualitative standards than on quantitative ones (cf. Wirth and Leutner, 2008). Moreover, by reproducing knowledge about beneficial and adverse strategies in these situations and comparing it to expert answers about those strategies, it overcomes a mere self-assessment. When it comes to the “action” part, about whether students really apply the beneficial strategies, it cannot overcome biases such as socially desirable answers. However, we belief that by first being able to show off that you know a strategy would be beneficial or adverse, it is easier to admit right afterwards that you do not always apply it. After all, the discrepancies we find between knowledge and action, underline this argument. If students would have answered in a socially desirable way, there would be no or at least just a small difference between knowledge and action.

In an attempt to assess students' actions in a more ecologically valid way, we decided to use a dichotomous answering format, since a graduation of “using a strategy” or “not using a strategy” seemed counterintuitive. Concerning the sub-samples resulting from the adaptive format, it must also be mentioned that the aspired parallelization of the sample could not be achieved on the sub-sample level. Since the parallelization of Bachelor and Master students of psychology and economic sciences could not be achieved, it was not possible to compare less experienced with more experienced students. Therefore, this study represents a first exploratory step toward understanding discrepancies between knowledge and action and to understanding what prevents us from using SRL strategies. The descriptive results have to be interpreted with caution. In further research, the answering format as well as the adaptive process should be changed in order to get interval data of bigger subsamples for being able to overcome these limitations.

To overcome the discrepancy between knowledge and action, interventions must focus on procedural knowledge (how to use a certain SRL strategy), conditional knowledge (when to choose which strategy) and most of all “hands-on practice” (Weinstein et al., 2000). This study makes it very clear that we cannot stop at the level of knowledge transfer but must guide students in the phase of applying what they have learned, promote the usage of what they have learned and help them differentiate their strategy usage depending on the task at hand. Research should shift its focus from the mere operationalization of SRL competence indicators to a more holistic view and a more differentiated understanding of strengths and weaknesses. Future research should dig deeper into these mechanisms and turn its focus to triangulated intervention programs so as to enable economical large-scale assessments that go beyond offline quantitative standards. To ensure practice-oriented implementation, the mechanisms behind the SRL competence scores of various measurements need to be better understood. Much as students possess broad SRL knowledge but are not able to put it into practice, research itself also still needs to bridge the gap between knowledge and action. Without taking the situation specificity inherent in the construct of competence into consideration and linking the shifting relevance and fit of SRL strategies to higher education challenges, research cannot substantially contribute to evidence-based implementation. With regard to the very high rates of knowledge exhibited by the sample in this questionnaire, it should be mentioned that “knowing” in the context of this study consists of recognizing and correctly identifying a strategy. The students in our sample might have actually known a lot about SRL strategies, or the identification of beneficial strategies in this questionnaire might have been too easy. However, social desirability does not seem to have confounded the answers, since a discrepancy between knowledge and use is apparent. It can be assumed that if social desirability bias had been relevant, the proclaimed usage of strategies would have been equivalently high. Nevertheless, a study with a non-academic sample could provide some insightful information about whether students truly know as much about SRL strategies as indicated by the results of this study.

### Conclusion

The most prominent result of our study lies in the striking discrepancies between SRL knowledge and action among university students. Our results indicate that students possess a considerable amount of knowledge regarding SRL strategies throughout their study programs. However, students' SRL competences seem to be confined to knowing about SRL strategies, as they often do not manage the transfer into action that is necessary to benefit from this knowledge. In order to understand these discrepancies, this study attempts to locate students' self-reported reasons within the theories of mediation and production deficiencies as well as value-expectancy assumptions.

## Ethics statement

This study was carried out in accordance with the recommendations of the ethical standards provided by the Austrian Federal Ministry of Health (Bundesministerium fuer Gesundheit, 1995) and the American Psychological Association [APA] (2010). Ethical approval was not required for this study in accordance with the national and institutional guidelines.

## Author contributions

NF: Data analysis, manuscript preparation and final writing. JK: Study design, manuscript preparation and final writing. GJ: Study design and data collection. CS and BS: Supervision, review and final approval.

### Conflict of interest statement

The authors declare that the research was conducted in the absence of any commercial or financial relationships that could be construed as a potential conflict of interest.
