# Supplementary material for: Knowledge vs. Action: Discrepancies in University Students' Knowledge about and Self-Reported Use of Self-Regulated Learning Strategies
Source: Front Psychol. 2017 Jul 27;8:1288. doi: 10.3389/fpsyg.2017.01288 (PMC5529389; doi:10.3389/fpsyg.2017.01288)
Supplement: Supplementary file 1 [file Appendix.PDF]

Appendix A: Vignettes (compare Dresel et al., 2015).

Frustration during the process of working on a major scientific thesis

Imagine you are working on a major scientific thesis in your course of studies (Diploma Thesis, Master Thesis, Bachelor Thesis, potentially). For this purpose, a broad topic must be processed self-reliantly over several months under supervision of a professor.

The results of a survey conducted with students has shown frustration to be a central problem in this process.

From your perspective, what do you think is beneficial to avoid frustration during the process of working on a major scientific thesis?

What do you actually do, to prevent yourself from getting frustrated while working a major scientific thesis?

Writing a minor scientific work

Imagine you are working on a minor scientific work in your course of studies, meaning a scientific text which requires you to constantly research and process literature and must be handed in at a fixed date (seminar paper, essays, e.g.).

The results of a survey conducted with students has shown it is of great importance to reflect on your approach on this task.

From your perspective, what do you think is beneficial while writing a minor scientific work?
What do you actually do when you are writing a minor scientific work?

Exam Preparation

Imagine you are preparing for an exam. You must process and learn large quantities of a subject matter autonomously whereas the profound understanding of the subject matter is essential.

The results of a survey conducted with students has shown learning strategies to be highly important in this situation.

From your perspective, what do you think is beneficial while preparing for an exam?

What do you actually do while preparing an exam?

Boredom during a lecture

Imagine you are attending a lecture, meaning a course in which a docent presents a large body of study matter in front of a rather large number of students.

The results of a survey conducted with students has shown that boredom is often a problem. From your perspective, what do you think is beneficial to avoid boredom during lecture attendance?

What do you actually do to prevent boredom during lecture attendance?

This is a provisional file, not the final typeset article
